# Sensor Management Method of Giving Priority to Confirmed Identified Targets

**DOI:** 10.3390/s23083959

**Published:** 2023-04-13

**Authors:** Chunshan Ding, Chunguo Li

**Affiliations:** 1 School of Information Science and Engineering, Southeast University, Nanjing 210096, China; 2 Jiangsu Automation Research Institute, Lianyungang 222006, China

**Keywords:** sensor management, desired confidence level, target identification, information theory, entropy

## Abstract

The optimization objective function of sensor management for target identification is commonly established based on information theory indicators such as information gain, discrimination, discrimination gain, and quadratic entropy, which can control the sensors to reduce the overall uncertainty of all targets to be identified but ignores the speed of target being confirmed as identified. Therefore, inspired by the maximum posterior criterion of target identification and the target identification confirmation mechanism, we study a sensor management method that preferentially allocates resources to identifiable targets. Firstly, in the distributed target identification framework based on Bayesian theory, an improved identification probability prediction method that provides feedback the global identification results to local classifiers is proposed, which can improve the accuracy of identification probability prediction. Secondly, an effective sensor management function based on information entropy and expected confidence level is proposed to optimize the identification uncertainty itself rather than its variation, which can increase the priority of targets that satisfy the desired confidence level. In the end, the sensor management for target identification is modeled as a sensor allocation problem, and the optimization objective function based on the effective function is constructed, which can improve the target identification speed. The experimental results show that the correct identification rate of the proposed method is comparable to the methods based on information gain, discrimination, discrimination gain, and quadratic entropy in different scenarios, but the average time to confirm the identification is the shortest.

## 1. Introduction

**Figure 1:**
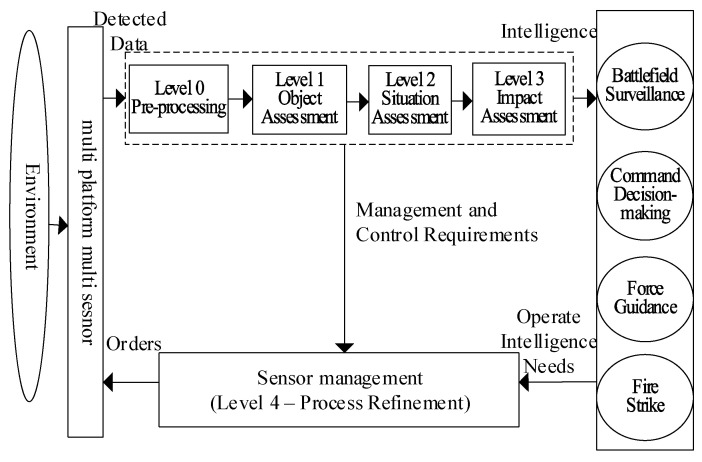
Sensor management constitutes information fusion feedback control.

According to the information fusion process functional model of the joint board of the laboratory of the United States Department of Defense, target identification in layer 1 fusion object estimation extracts abstract target features and attributes from direct sensor measurement data and provides the basis for its estimated target classification. Target recognition is a very important, but difficult to achieve, function in information fusion. In order to make up for the deficiency of information fusion open-loop processing, sensor management function is introduced in the fourth layer of fusion process refining. Specifically, according to the processing results from level 0 to level 3, the future sensor usage scheme is used to achieve application goals such as early target detection, improved track quality, accurate target identification, and evidence collection for tactical decision making; therefore, allowing for information fusion with a feedback loop as shown in Figure 1 [1]. The purpose of sensor management is to utilize limited sensor resources in such a way that they detect and acquire the input data required for back-end information processing to meet the information requirements of applications such as weapon strikes and command and control. Sensor management is achieved by determining the parameters of the sensor’s degrees of freedom through optimal criteria while also satisfying practical operational constraints [1].

The problem of sensor management in target recognition has been extensively studied by scholars. Yang et al. summarized sensor management methods in target recognition [2]. Similar to the general sensor management problem, the sensor management oriented to target recognition is mainly solved based on optimization technology. The research contents mainly include problem modeling and optimization objective design. In terms of problem modeling, sensor management is usually modeled as linear programming, or as sequential decision problem, such as Markov decision process (MDP), or partially observable Markov decision process (POMDP). The research is focused on how to solve the POMDP problem [3,4,5,6,7,8,9,10]. In the aspect of optimization objective design, the information theory method is widely used in sensor management because it can describe the uncertainty of target motion state estimation and target identification. For example, Rényi entropy and Cauchy–Schwartz (CS) divergence are used in sensor management of target tracking [11,12,13]. In sensor management for target recognition, information theory is also widely used to construct the optimal criterion of sensor management, and a series of achievements have been achieved. In terms of discrimination gain, Kastella proposed the method of selecting sensors with the discrimination gain expectation of the target recognition probability as the optimization objective for target detection and recognition problem [14]. Jenkins also proposed a method to establish an optimal objective function for sensor management based on the discrimination gain expectation, which is used to solve the adaptive management problem of image sensors for target classification, proposing the use of Bhattacharyya coefficients and Chernoff coefficients for offline estimation of the quantitative likelihood function between different hypotheses [15,16]. Kolba applied the sensor management approach from the literature [14] to detect and identify mines and extended the algorithm to different application scenarios such as multi-target, multi-sensor platforms, and multi-mode sensors [17]. The literature [18] proposes a predictive discrimination gain calculation method using the sensor confusion matrix. In terms of discrimination, the literature [19] uses D-S evidence theory for target identification inference, which is based on the principle of making the system predictive identification discrimination maximum for sensor optimization management. In terms of information gain, [20] proposes a search strategy for sensors based on maximum information gain for discrete detection units, which implements a sensor management algorithm for detection and classification. The authors of [21] used Bayesian networks to obtain multi-sensor feature-level cooperative sensing probabilities. The method establishes a closed-loop control from cooperative target identification to dynamic management of sensors based on the entropy gain of joint sensing information and uses an intelligent optimization algorithm to improve the efficiency of the “multi-sensor and multi-target” assignment. In a fused target recognition system based on D-S evidence theory, [22] proposed two methods of sensor–target assignment with the predicted information gain, and the predicted cumulative uncertainty assigned to the recognition frame as the optimization objectives, respectively. The authors of [23] compared the performance of several information-theoretic-based search strategies and decision rules for a typical target classification problem. Numerous numerical simulations have shown that the standard quadratic entropy as the correct classification rate is usually the most effective search strategy. Some scholars have studied the sensor management method of integrated target tracking and recognition [24,25,26,27] and proposed the optimization objective function based on the threat or risk [24,25].

In the actual target recognition application, the identification–confirmation mechanism is generally adopted, that is, when the probability of the target belonging to a certain category is greater than the identification–confirmation threshold, the identification of the target is considered to have been completed, and the subsequent identification and judgment can be terminated [2,23]. Only the result of confirmation–identification is considered credible, and response measures can be taken against the target according to the identification result. Therefore, for users of identification results, it is very important to confirm the identification of targets as soon as possible. We study the sensor management method to improve the speed of target recognition–confirmation, which is a novel method based on task-driven, and there is no relevant research in the current literature. Information theory is the theoretical basis of uncertainty, which has been widely used in the research of sensor management for target recognition, so this paper is also based on information theory.

Using the information theory method to manage sensors can improve the correct target identification rate, but it does not focus on the problem of target identification confirmation speed. The aim of sensor management for target identification based on an information theory-based approach is to reduce the overall uncertainty of all targets to be identified. However, the above methods are not considered to match the identification confirmation mechanism. Information entropy is a quantitative description of uncertainty. Information entropy cannot correctly reflect the amount of information. It is the change of information entropy that generates information. Information gain and discrimination are both information measures reflecting the change of information entropy, while discrimination gain is a measure to obtain the change of information quantity. Therefore, the aim of the information gain and discrimination-based approach to sensor management is to maximize the amount of information acquired by detection, i.e., the sum of all target information entropy reduction values. The objective of the discrimination gain-based approach to sensor management is to maximize the increase in the amount of detected information. However, since the amount of information acquired by further detection of targets with high recognition probability is relatively small, the above methods are not conducive to prioritizing such targets to meet the recognition confirmation threshold, resulting in a lack of recognition confirmation speed. Take the example of a sensor detecting two targets. The target categories are two categories. At the same time, one sensor can only observe one target. The current recognition state probability distributions of target 1 and target 2 are $\pi_{1}\left(t\right)=\left(0.87,0.13\right)$, $\pi_{2}\left(t\right)=\left(0.5,0.5\right)$, the recognition confirmation threshold is set to 0.9. From the perspective of fast confirmation of target identification, target 1 is further detected by using the sensor to increase the recognition probability to the recognition confirmation threshold, thus confirming that target 1 has been identified. The reason for using the sensor for target 2 detection based on information gain, discrimination, etc., is that the information obtained for target 2 detection is greater than that for target 1. However, in this case, neither target can be confirmed for identification. Clearly, identification of target 1 is the more reasonable sensor use decision. Aiming at the shortcomings of existing sensor management methods based on information theory in terms of target recognition confirmation speed, this paper proposes a new method. The newly proposed method combines the maximum posterior criterion of sensor management with the target recognition–confirmation mechanism of target recognition.

The method improves the target recognition probability prediction by feeding the global recognition results back to the local classifier to provide a more accurate recognition effectiveness estimation for sensor management. Additionally, a segmented target recognition effectiveness function based on information entropy and recognition confirmation threshold is constructed to optimize the uncertainty of the target recognition rather than the amount of variation, to increase the priority of targets that satisfy the recognition confirmation threshold, thus ensuring the correct recognition of targets while reducing the confirmation recognition time.

This paper is organized as follows: Section 2 introduces the sensor management problem in distributed target identification. Section 3 describes the proposed sensor management method for giving priority to confirmed identified targets. Section 4 illustrates the simulation experiment process and results, and Section 5 is the conclusion.

## 2. Sensor Management Issues in Distributed Target Recognition

To ensure the accuracy and reliability of target identification, it is often necessary to collect multi-dimensional feature information from multiple platforms and sensors, and then identify the target by fusing multiple information sources. In multi-platform distributed target recognition, a hierarchical decision-level target recognition structure is usually used, i.e., a local classifier first estimates the unknown target class based on the observations of the sensors and then aggregates this local recognition result to the fusion center, which makes a global recognition estimate based on the received multiple local recognition results. 

**Figure 2:**
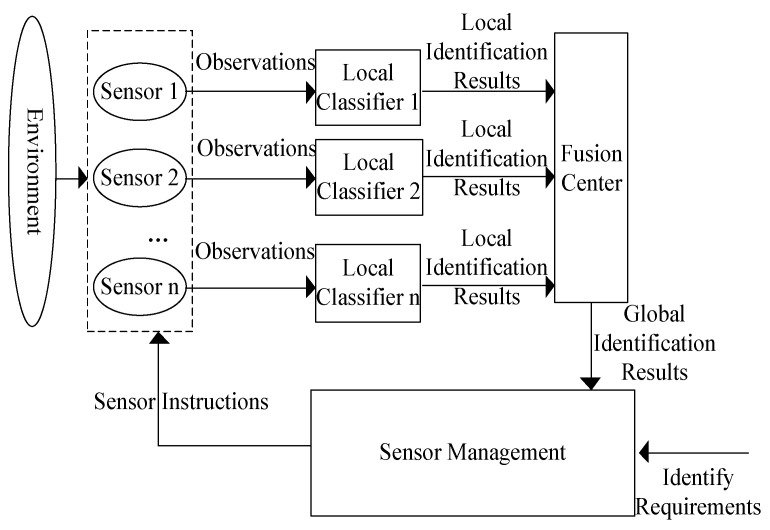
Target identification process with feedback.

The above target identification process is open-loop, and after the introduction of sensor management, a closed-loop target identification process with feedback is formed, as shown in Figure 2. After the sensor observes the target, the target recognition result is obtained through the distributed target recognition estimation, the sensor management module makes the next sensor control decisions based on the target recognition result and the recognition demand, and the feedback control sensor obtains the observation that better allows the recognition result to meet the recognition demand in order to improve the overall effectiveness of the target recognition.

### 2.1. Hierarchical Decision-Level Target Identification

Consider a scenario where the sensor network consisting of *R* sensors identifies *N* targets. The category of targets belongs to a known target identification domain Ψ with complete and mutually exclusive *M* elements, $\Psi ≜\left(\omega_{1},\omega_{2},\cdots ,\omega_{M}\right)$. Let $c_{i}$ denote the true category of target *i*, and $c_{i}$ is a fixed value that does not change with time but is unknown and needs to be estimated by sensor observations. Assume the category of target *i* is a random variable $x_{i}$, whose prior probability is known. The process of target identification at the hierarchical decision level is as follows.

(a) Sensor-level target identification

For the target *i*, the measurement of sensor *j* at the moment *t* is $z_{ij}\left(t\right)$, and all measurements obtained at time *t* for target *i* are $Z_{ij}\left(t\right)$, where $Z_{ij}\left(t\right)=\left(z_{ij}\left(0\right),\cdots ,z_{ij}\left(t\right)\right)$. The target classification result of the local classifier is $P(x_{i}|Z_{ij}\left(t\right))$, j=1,2,⋯, R, then, the local identification results are reported to the fusion center. If sensor *j* cannot detect target *i*, the corresponding measurement is empty, and no classification result is reported to the fusion center.

(b) Decision-level fusion for target identification

The optimal identification estimation of the target by the fusion center should be based on all sensor measurements $Z_{i}\left(t\right)=\left(Z_{i1}\left(t\right),\cdots ,Z_{iR}\left(t\right)\right)$. It is necessary to know the correlation between the measurements of the information sources in the observation set $Z_{i}\left(t\right)$, that is, the conditional probability knowledge of each sensor measurement $P(Z_{i1}(t),\cdots ,Z_{iR}(t)|x_{i}=\omega_{k})$. If the target category is assumed to be $\omega_{k}$, and sensor measurements are conditionally independent, i.e.,

(1) $$P(Z_{i1}(t),\cdots ,Z_{iR}(t)|x_{i}=\omega_{k})=\prod_{j=1}^{R}P(Z_{ij}(t)|x_{i}=\omega_{k})$$

Then, according to the Bayesian theory,

(2) $$P\left(x_{i}|Z_{i1}\left(t\right),\cdots ,Z_{iR}\left(t\right)\right)=\frac{P\left(Z_{i1}\left(t\right),\cdots ,Z_{iR}\left(t\right)|x_{i}\right)P\left(x_{i}\right)}{P\left(Z_{i1}\left(t\right),\cdots ,Z_{iR}\left(t\right)\right)}\;=\frac{P\left(x_{i}\right)\prod_{j=1}^{R}P\left(Z_{ij}\left(t\right)|x_{i}\right)}{P\left(Z_{i1}\left(t\right),\cdots ,Z_{iR}\left(t\right)\right)}$$

Since $P\left(Z_{ij}\left(t\right)|x_{i}\right)=\frac{P(x_{i}|Z_{ij}\left(t\right))P\left(Z_{ij}\left(t\right)\right)}{P\left(x_{i}\right)}$, $P\left(Z_{i1}\left(t\right),\cdots ,Z_{iR}\left(t\right)\right)=\sum_{v=\omega_{1}}^{\omega_{M}}P\left(Z_{i1}\left(t\right),\cdots ,Z_{iR}\left(t\right)|v\right)P\left(v\right)$, then

(3) $$P\left(x_{i}|Z_{i1}\left(t\right),\cdots ,Z_{iR}\left(t\right)\right)=\frac{P\left(x_{i}\right)\prod_{j=1}^{R}\left[\frac{P\left(x_{i}|Z_{ij}\left(t\right)\right)P\left(Z_{ij}\left(t\right)\right)}{P\left(x_{i}\right)}\right]}{\sum_{v=\omega_{1}}^{\omega_{M}}P\left(Z_{i1}\left(t\right),\cdots ,Z_{iR}\left(t\right)|v\right)P\left(v\right)}\;=\frac{P{\left(x_{i}\right)}^{-\left(R-1\right)}\prod_{j=1}^{R}\left[P\left(x_{i}|Z_{ij}\left(t\right)\right)P\left(Z_{ij}\left(t\right)\right)\right]}{\sum_{v=\omega_{1}}^{\omega_{M}}P{\left(v\right)}^{-\left(R-1\right)}\prod_{j=1}^{R}\left[P\left(v|Z_{ij}\left(t\right)\right)P\left(Z_{ij}\left(t\right)\right)\right]}\;=\frac{P{\left(x_{i}\right)}^{-\left(R-1\right)}\prod_{j=1}^{R}P(x_{i}|Z_{ij}\left(t\right))}{\sum_{v=\omega_{1}}^{\omega_{M}}P{\left(v\right)}^{-\left(R-1\right)}\prod_{j=1}^{R}P(v|Z_{ij}\left(t\right))}$$

where $P(x_{i}|Z_{ij}\left(t\right))$ is the probability of the target *i* belonging to category $x_{i}$ according to the measured $Z_{ij}\left(t\right)$ generated by the local classifier *j* and reported to the fusion center. This indicates that the fusion center can make target identification estimates based on local recognition results rather than raw sensor measurements, which is intended to reduce the amount of data reported by sensors to the fusion center.

After obtaining $P\left(x_{i}|Z_{i1}\left(t\right),\cdots ,Z_{iR}\left(t\right)\right)$, the maximum posterior criterion is used to determine. Let the target *i* recognition result be $\omega_{f}$, then

(4) $$\omega_{f}=argmax\left(P\left(x_{i}|Z_{i1}\left(t\right),\cdots ,Z_{iR}\left(t\right)\right)\right)$$

If $P\left(x_{i}=\omega_{f}|Z_{i1}\left(t\right),\cdots ,Z_{iR}\left(t\right)\right)>\varepsilon$, ε is the recognition confirmation threshold, then the recognition result of target *i* is considered to reach a confidence level without further observation and recognition [2,23], and the user can trust the recognition result. Additionally, if $P\left(x_{i}=\omega_{f}|Z_{i1}\left(t\right),\cdots ,Z_{iR}\left(t\right)\right)<\varepsilon$, then the recognition result is fuzzy, which therefore cannot determine the target category and needs to continue the recognition process.

### 2.2. Sensor Management for Target Recognition

The sensor management of discrete-time target recognition is the decision-making about the next moment of sensor action, where the sensor action is simplified to whether or not to detect a certain target. It is decided at time t which targets are detected by the sensors, and the sensor detect action $u_{ij}\left(t\right)$ is defined as:

(5) $$u_{ij}\left(t\right)=\{\begin{array}{c} 1\;\;\;\mathrm{Sensor}\;j\;\mathrm{detects}\;\mathrm{target}\;i\;\mathrm{at}\;\mathrm{time}\;t\\ 0\;\;\;\mathrm{Others} \end{array}$$

Then, the sensor network detection action at time *t* for target *i* is $u_{i}\left(t\right)=\left(u_{i1}\left(t\right),\cdots ,u_{iR}\left(t\right)\right)$. The *j*-th sensor $s_{j}$ in sensor set S detects target *i* and obtains an observation $z_{ij}\left(t\right)$, whose probability $p(z_{ij}\left(t\right)|s_{j},c_{i})$ is known and independent of the sampling instants. 

The conditional probability distribution of the state $x_{i}$ of target *i* is $\pi_{i}\left(t\right)=P[x_{i}|I\left(t\right)]$. Since the targets are independent of each other, let $\pi_{ik}\left(t\right)$ denotes the probability of $x_{i}=\omega_{k}$ under the conditions of information I(t) has been obtained, then

(6) $$\pi_{i}\left(t\right)=\left(\pi_{i1}\left(t\right),\pi_{i2}\left(t\right),\cdots ,\pi_{iM}\left(t\right)\right)$$

Since the initial probability of the target category is known, $I\left(0\right)=\left\{\pi_{i}\left(0\right),i=1,\ldots ,\;N\right\}$, the information at time *t* is

(7) $$I\left(t\right)=I\left(t-1\right)\cup \left\{u_{i}\left(t\right),z_{i}\left(t\right),i=1,\ldots ,N\right\}$$

Thus, the state probability distributions, measurements, and sensor detection actions of all targets can be obtained as follows:

(8) $$\vec{\pi }\left(t\right)=\left(\pi_{1}\left(t\right),\cdots ,\pi_{N}\left(t\right)\right)\;\vec{z}\left(t\right)=\left(z_{1}\left(t\right),\cdots ,z_{N}\left(t\right)\right)\;\vec{u}\left(t\right)=\left(u_{1}\left(t\right),\cdots ,u_{N}\left(t\right)\right)$$

The problem that sensor management needs to solve is to determine sensor actions at the next moment $\vec{u}\left(t+1\right)$ based on I(t) and $\vec{\pi }\left(t\right)$, It can be modeled as a problem of assigning sensors to targets to be identified.

Since a sensor can observe multiple targets at the same moment, it is also possible to assign multiple sensors to observe the same target, and the sensors in the sensor set S are called basic sensors. The pseudo-sensor is a synthetic sensor composed of a set of basic sensors, such that at most one sensor (basic sensor or pseudo-sensor) is assigned to a target at the same moment.

The sensor $k\left(k=1,2,\cdots ,2^{R}-1\right)$ assigned to the target i(i=1,2,⋯, N) is denoted as $e_{ij}=1$, otherwise $e_{ij}=0$, then all $e_{ij}$ formed assignment matrix E of the $N\times (2^{R}-1)$ order. Let the maximum number of simultaneous measurement targets of basic sensor *j* be $l_{j}\left(j=1,2,\cdots ,\;R\right)$, and S(j) be an integer set composed of the serial numbers of all sensor combinations (basic sensors and pseudo-sensors) including basic sensor *j*. Then, the sensor management for target identification can be defined as the following allocation problem in the sense of expecting the sum of detection effectiveness of each sensor for the target under the sensor usage decision to be maximum.

(9) $$E=\underset{E}{\mathrm{argmax}}(\overset{2^{R}-1}{\underset{k=1}{\sum }}\overset{N}{\underset{i=1}{\sum }}q_{ik}e_{ik})$$

where the $q_{ik}$ is the sensor effectiveness function for target detection, which directly affects subsequent sensor usage decisions and is the key to modeling the sensor management problem. Information gain, discrimination, discrimination gain, and quadratic entropy are all methods commonly used to construct the effectiveness function based on information theory, as described in Appendix A.

Additionally, we satisfy the following constraints:

(10) $$\{\begin{array}{c} \sum_{k=1}^{2^{R}-1}e_{ik}\leq 1,\;i=1,2,\cdots ,N\\ \sum_{k\in S\left(j\right)}e_{ik}\leq l_{j},j=1,2\cdots ,R \end{array}$$

The purpose of the first constraint is to limit the recognition of the same target by no more than one sensor (combination), and the purpose of the second constraint is to limit the number of targets assigned to a sensor to no more than the maximum number of detected targets.

## 3. Sensor Management Method of Giving Priority to the Confirmable Identify Targets

To improve the speed of target recognition, this paper proposes a sensor management method that gives the priority to the confirmed identified targets. The proposed method makes improvements in both recognition result prediction and effectiveness function construction. First of all, sensor management is the sensor assignment in the next moment. The prediction accuracy of the target recognition result by the sensors in the next moment directly affects the effectiveness of the sensor management decision, which is the basis for improving the speed of target recognition, for which the global recognition result is fed back to the local classifier to improve the target recognition probability prediction. Secondly, in order to fit with the maximum posterior criterion and the target recognition confirmation mechanism, the information entropy, which is more conducive to optimizing the probability distribution of the target category, is selected to construct the target recognition effectiveness function and to increase the priority of confirmable recognition targets. Finally, based on the allocation constraint function and objective function of the sensor, the 0–1 integer programming is used to solve the sensor action and realize the sensor management method of allocating resources to the confirmable recognition target with priority.

### 3.1. Global Fusion Recognition Results Feed Local Classifier for Target Recognition Prediction

For the target *i*, if the sensor *j* can predict the identification result $x_{i}(t|t-1)$ by using the measurement set $Z_{ij}\left(t-1\right)$ obtained until the time *t* − 1 before the measurement $z_{ij}\left(t\right)$ is obtained by the sensor *j* at the time *t*, according to [14]

(11) $$P\left(x_{i}|Z_{ij}\left(t\right)\right)=\frac{P\left(z_{ij}\left(t\right)|x_{i}\right)P\left(x_{i}|Z_{ij}\left(t-1\right)\right)}{\sum_{v=w_{1}}^{w_{M}}P\left(z_{ij}\left(t\right)|v\right)P\left(v|Z_{ij}\left(t-1\right)\right)}$$

Since $z_{ij}\left(t\right)$ is not actually obtained at time *t* − 1 and is unknown, if the measurement is discrete-valued, then the probability distribution of $z_{ij}\left(t\right)$ is as follows:

(12) $$P\left(z_{ij}\left(t\right)|Z_{ij}\left(t-1\right)\right)=\sum_{v=w_{1}}^{w_{M}}P\left(z_{ij}\left(t\right)|v\right)P(v|Z_{ij}\left(t-1\right))$$

The expected value of the probability distribution of the predicted recognition result at moment *t* is $E\left(P\left(x_{i}|Z_{ij}\left(t|t-1\right)\right)\right)$.

(13) $$E\left(P\left(x_{i}|Z_{ij}\left(t|t-1\right)\right)\right)=\sum_{z_{ij}\left(t\right)}P\left(x_{i}|Z_{ij}\left(t\right)\right)P\left(z_{ij}\left(t\right)|Z_{ij}\left(t-1\right)\right)\;=\underset{z_{ij}\left(t\right)}{\sum }\frac{P\left(z_{ij}\left(t\right)|x_{i}\right)P\left(x_{i}|Z_{ij}\left(t-1\right)\right)}{\sum_{v=w_{1}}^{w_{M}}P\left(z_{ij}\left(t\right)|v\right)P\left(v|Z_{ij}\left(t-1\right)\right)}\overset{w_{M}}{\underset{v=w_{1}}{\sum }}P\left(z_{ij}\left(t\right)|v\right)P\left(v|Z_{ij}\left(t-1\right)\right)\;=\underset{z_{ij}\left(t\right)}{\sum }P\left(z_{ij}\left(t\right)|x_{i}\right)P\left(x_{i}|Z_{ij}\left(t-1\right)\right)=P\left(x_{i}|Z_{ij}\left(t-1\right)\right)$$

It can be seen from Equation (13) that the expected value of the probability distribution of the predicted recognition result of a single sensor is the estimated value at the previous moment, and only its detection information is used in the prediction. Recognition result prediction is the basis for sensor assignment decisions, and more accurate prediction is conducive to more reasonable sensor assignment, which in turn improves recognition speed. Therefore, we propose to use the detection information of other sensors to improve the recognition result prediction of a single sensor, i.e., the global fusion recognition result is fed back to the local classifier, and the local classifier uses the fusion center recognition result to predict the recognition probability distribution of the sensor. Let the fusion center identification result be $\omega_{f}$, then the predicted observation value $P(z_{ij}\left(t\right))$ =$\;P\left(z_{ij}\left(t\right)|\omega_{f}\right)$ of sensor *j* at time *t* is the possible detection result when the target category is $\omega_{f}$.

(14) $$\overset{´}{E}\left(P\left(x_{i}|Z_{ij}\left(t|t-1\right)\right)\right)=\underset{z_{ij}\left(t\right)}{\sum }P\left(x_{i}|Z_{ij}\left(t\right)\right)P\left(z_{ij}\left(t\right)|\omega_{f}\right)$$

In (14), $P\left(x_{i}|Z_{ij}\left(t\right)\right)$ can be calculated recursively according to (11), and $P\left(z_{ij}\left(t\right)|\omega_{f}\right)$ is a known item, that is $p(z_{ij}\left(t\right)|s_{j},\;c_{i})$, where $c_{i}=\omega_{f}$. Denote the predicted value of the recognition probability distribution of the basic sensor *j* as $\vec{P}\left(x_{i}|Z_{ij}\left(t|t-1\right)\right)$, $\vec{P}\left(x_{i}|Z_{ij}\left(t|t-1\right)\right)=\overset{´}{E}\left(P\left(x_{i}|Z_{ij}\left(t|t-1\right)\right)\right)$.

Taking $\overset{´}{E}\left(P\left(x_{i}{|Z}_{ij}\left(t|t-1\right)\right)\right)$ as $P(x_{i}|Z_{ij}\left(t\right))$, then the predicted target recognition probability distribution $\vec{P}\left(x_{i}|Z_{ik}\left(t|t-1\right)\right)$ of the pseudo-sensor (sensor combination) can be calculated based on the Bayesian combination classification method; *k* is the number of the pseudo-sensor.

### 3.2. Efficacy Function Based on Information Entropy and Confirmed Identification Threshold

The identification result $\vec{P}\left(x_{i}|Z_{ik}\left(t|t-1\right)\right)$ of the target *i* by the *k*-th sensor (basic sensor or pseudo-sensor) at time *t* can be predicted. Let the maximum probability in the identification result be ${\vec{P}}_{max}\left(x_{i}\right)$.

(15) $${\vec{P}}_{max}\left(x_{i}\right)=\underset{x_{i}}{\max}[\vec{P}(x_{i}|Z_{ik}\left(t|t-1\right))]\;k=1,2,\cdots ,2^{R}-1$$

Define the effectiveness function of sensor *k* on target *i* as $q_{ik}$, and the classification confirmation degree threshold is ε. The effectiveness function based on information entropy and recognition confirmation threshold is constructed as Equation (16).

(16) $$q_{ik}=\{\begin{array}{c} \begin{array}{cc} \;\;\;\;\;\;\;\;\;\;\;\;\;\;\;\;\;\;\;\;1\;\;\;\;\;\;\;\;\;\;\;\; & \;\;\;\;\;{\vec{P}}_{max}\left(x_{i}\right)\geq \varepsilon \end{array}\;\\ \begin{array}{cc} 1-\frac{H\left(\vec{P}\left(x_{i}|Z_{ik}\left(t|t-1\right)\right)\right)}{H_{max}} & {\vec{P}}_{max}\left(x_{i}\right)<\varepsilon \end{array} \end{array}$$

where $H\left(\vec{P}\left(x_{i}|Z_{ik}\left(t|t-1\right)\right)\right)$ is the entropy of $\vec{P}\left(x_{i}|Z_{ik}\left(t|t-1\right)\right)$, and $H_{max}$ is the entropy when the probability of each category is equal. Information entropy is used here because its quantitative representation of uncertainty in target identification is directly related to the prediction of maxima probability in identification results. Lower entropy means less uncertainty and a higher probability maximum in the prediction recognition result. When the probability distribution of the target belonging to each category is uniform, the information entropy is the largest, the maximum probability in the prediction recognition result is the smallest, and the uncertainty is also the largest.

The information gain, discrimination, and discrimination gain reflect the relative change amount, which is not directly related to the probability maximum in the prediction recognition result, and therefore the optimization ability of the probability maximum in the prediction recognition result is not as good as the information entropy. ${\vec{P}}_{max}\left(x_{i}\right)\geq \varepsilon$, $c_{ik}$ is directly assigned to be 1 in order to improve the priority of meeting the classification confirm threshold.

### 3.3. Target Identification Process Integrated into Sensor Management

**Figure 3:**
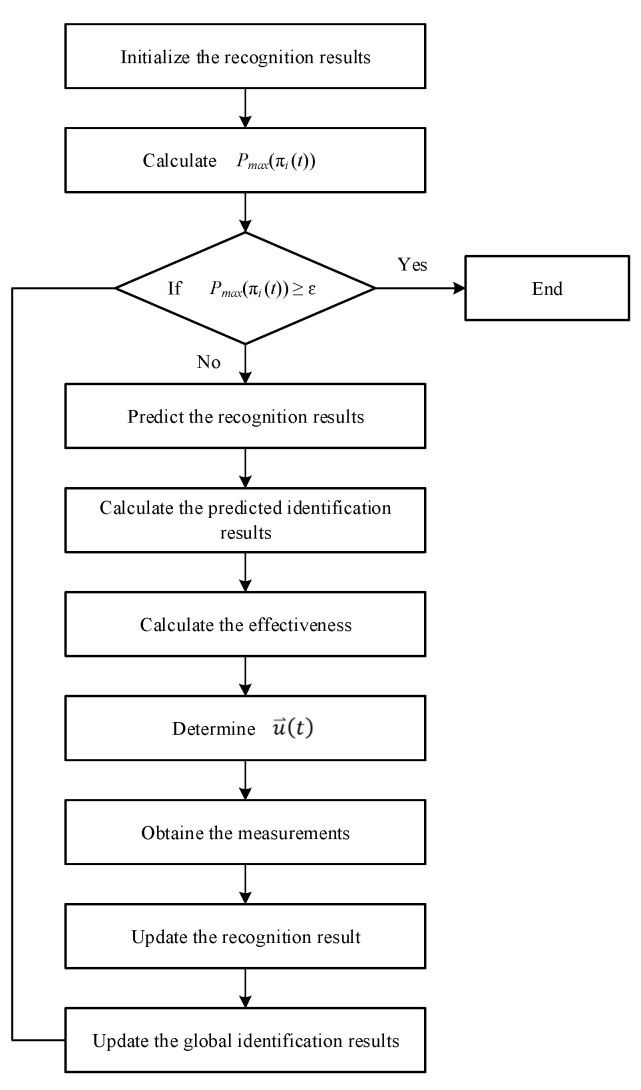
The flow chart of the proposed method.

In summary, the sensor management processes is introduced to construct a distributed target recognition process with feedback, the flow chart of the proposed method is shown in Figure 3 and the specific processing steps are as follows.

Step 1. Initialize the recognition results of each basic sensor for the target and the recognition results of the fusion center.

Let t=0, and the target recognition result of the initialized basic sensor *j* is ${\vec{\pi }}^{j}\left(0\right)=\left(\pi_{1}^{j}\left(0\right),\cdots ,\pi_{N}^{j}\left(0\right)\right)$, j=1,2,⋯, R, where $\pi_{1}^{j}\left(0\right)=\left(\pi_{i1}^{j}\left(0\right),\pi_{i2}^{j}\left(0\right),\cdots ,\pi_{iM}^{j}\left(0\right)\right)$, i=1,2,⋯, N is the known prior probability of the target category.

Step 2. Identification confirmation. For target *i*, the maximum probability that the target belongs to a certain category in the recognition result $from\;P_{max}\left(\pi_{i}\left(t\right)\right)=max\left(\pi_{i1}\left(t\right),\pi_{i2}\left(t\right),\cdots ,\pi_{iM}\left(t\right)\right)$, if $P_{max}\left(\pi_{i}\left(t\right)\right)\geq \varepsilon$, then the identification of this target is terminated. ε is the recognition confirmation threshold.

If all targets to be identified have been confirmed for identification, the target identification process is terminated.

Step 3. Sensor management.

(1) Predict the recognition results of the basic sensors according to Equation (14).

(2) Calculate the predicted identification results of the pseudo-sensor according to Equation (3).

(3) Calculate the effectiveness of the basic sensor and pseudo-sensor according to Equation (16).

(4) The effectiveness function is substituted into the assignment problem defined by Equations (9) and (10), and the sensor action $\vec{u}\left(t\right)$ is determined using the 0–1 integer programming solution method.

Step 4. After sensor detection of the target, t=t+1, measurements $z_{ij}\left(t\right)$, i=1, 2,⋯, N, j=1, 2,⋯, R can be obtained.

Step 5. Each sensor updates the recognition result according to Equation (11) and reports it to the fusion center.

Step 6. The fusion center updates the global identification results according to Equation (3).

Then, return to step 2.

## 4. Simulation Results

### 4.1. Design of the Verification Method for the Proposed Algorithm

The operation processes of the methods based on information gain, discrimination, discrimination gain, and quadratic entropy are basically the same as the method in this paper, the difference is that the effectiveness function in sensor management as $q_{ij}$ is solved according to the definitions of information gain, discrimination, discrimination gain, and quadratic entropy in the appendix, and the recognition results of the basic sensor are predicted according to Equation (13).

The proposed method is compared with the methods based on information gain, discrimination, discrimination gain, and quadratic entropy. In step 2 of the identification confirmation process in Section 3.3, if the probability that target *i* belongs to a class is greater than or equal to the recognition confirmation threshold, then the recognition process for that target is ended. The recognition confirmation time of target *i* is $t_{i}$. If the final identified target category of target *i* is the same as the true category $c_{i}$, it is judged to be correctly identified and the number of correctly identified targets $N_{c}$ is added to 1. When all targets have been confirmed for identification, statistics of the correct recognition rate $P_{c}$ of this simulation and the average confirmation recognition time is $T_{a}$.

(17) $$T_{a}=\frac{1}{N}\sum_{i=1}^{N}t_{i}$$

(18) $$P_{c}=\frac{N_{c}}{N}*100\%$$

where N is the total number of simulated targets, and $N_{c}$ is the total number of correctly identified targets.

In the simulation validation, step 4 in Section 3.3 of the previous section is based on the detection performance of the sensor, i.e., according to the distribution probability $p\left(z_{ij}\left(t\right)|s_{j},c_{i}\right)$ of the observation information $z_{ij}\left(t\right)$ of the object *i* based on sensor *j*, the simulated data are obtained. $z_{ij}\left(t\right)$ is defined as the recognition result of the sensor on the target category instead of the original measurement. $p\left(z_{ij}\left(t\right)|s_{j},\;c_{i}\right)$ indicates the probability that the sensor *j* recognizes the target of real class $c_{i}$ as class $\omega_{k}$, which is called the confusion probability and can be obtained based on the statistical analysis of the sensor’s historical detection data. Therefore, the confusion matrix is constructed to characterize the characteristics of the sensor. The confusion matrix of sensor j is expressed as $P^{j}={\left(p_{kl}^{j}\right)}_{M\times M}$, element $p_{kl}^{j}$ is the probability that sensor *j* identifies the target of class *k* as the target of class *l* [18]. In practical applications, the measurement probability of a sensor may not only be related to the real category of target, but also to the specific situation and environment. It is necessary to model the detection performance according to the specific sensor model, so that the measurement probability varies with changes in situational and environmental information.

### 4.2. Design of the Scene

In many target recognition problems in military applications, the number of target types is less than five. For example, when it is necessary to judge the authenticity of the target, whether there is a target, and whether the target is a surface target or an air target, the number of target types can be considered to be two. When it is necessary to determine whether the surface target is a large ship, medium ship, or small ship, or whether the underwater target is a submarine, torpedo, or mine, the number of target types can be considered to be three. When it is necessary to determine whether the target attribute is enemy, our side, friend side, neutral, or unknown, the number of target types can be considered to be five. There are not many sensors to perform above identification tasks on platforms such as ships, aircraft, unmanned boats, and other unmanned platforms.

Considering the above application requirements, and to verify the sensor management efficiency of the proposed method in various situations of single sensor identification and multi-sensor cooperative identification, the following five scenarios are designed according to the number of target categories and applicable sensors. It is assumed that the surveillance areas in all scenarios consist of *N* = 10 discrete units and each unit has one target. Each sensor can only detect one target at a time.

The setting of scenario 1 focuses on clearly demonstrating the processing process of sensor management but does not involve multi-sensor cooperative identification. Scenarios 2–5 involve the collaborative recognition of multiple types of targets by multiple sensors, which can verify the decision-making level fusion by fusion center using multi-sensor identification information under hierarchical distribution, as well as the prediction process of multi-sensor recognition result of the same target in sensor management. The simulation results of five scenarios and five parameter configurations demonstrate that the proposed method can be applied to the recognition of multi class targets by multiple sensors. When there are more types and numbers of targets, the efficiency of sensor management depends on the efficient solution of the efficiency function constructed in this article, which is not the problem solved in this paper.

Scenario 1: Since it is detecting whether there is a target in each unit, then the number M of target types is 2, and only one sensor is used for target detection. When the confusion matrix is set to a fixed value, the confusion matrix is set according to the detection rate and false alarm rate of the sensor as follows:

$$P^{1}=\left[\begin{array}{cc} 0.87 & 0.13\\ 0.13 & 0.87 \end{array}\right]$$

Scenario 2: Identify the class of targets within each unit with type number M of 3. Two sensors are used to detect the targets. When the confusion matrix is set to a fixed value, confusion matrices are as follows:

$$P^{2}=\left[\begin{array}{ccc} 0.98 & 0.1 & 0.1\\ 0.01 & 0.8 & 0.1\\ 0.01 & 0.1 & 0.8 \end{array}\right]\;P^{3}=\left[\begin{array}{ccc} 0.8 & 0.1 & 0.01\\ 0.1 & 0.8 & 0.01\\ 0.1 & 0.1 & 0.98 \end{array}\right]$$

Scenario 3: Identify the class of the targets in each unit with type number M of 3. Three sensors are used to detect the targets. In addition to using the sensor in scenario 2, add another sensor with the following confusion matrix. When the confusion matrix is set to a fixed value, the confusion matrix of the added sensor is as follows:

$$P^{4}=\left[\begin{array}{ccc} 0.8 & 0.01 & 0.1\\ 0.1 & 0.97 & 0.1\\ 0.1 & 0.02 & 0.8 \end{array}\right]$$

Scenario 4: Identify the class of targets within each unit with type number M of 5. Two sensors are used to detect the targets. When the confusion matrix is set to a fixed value, confusion matrices are as follows:

$$P^{5}=\left[\begin{array}{ccccc} 0.96 & 0.05 & 0.02 & 0.04 & 0.05\\ 0.01 & 0.80 & 0.02 & 0.04 & 0.05\\ 0.01 & 0.05 & 0.92 & 0.04 & 0.05\\ 0.01 & 0.05 & 0.02 & 0.84 & 0.05\\ 0.01 & 0.05 & 0.02 & 0.04 & 0.80 \end{array}\right]\;P^{6}=\left[\begin{array}{ccccc} 0.80 & 0.04 & 0.05 & 0.02 & 0.01\\ 0.05 & 0.84 & 0.05 & 0.02 & 0.01\\ 0.05 & 0.04 & 0.80 & 0.02 & 0.01\\ 0.05 & 0.04 & 0.05 & 0.92 & 0.01\\ 0.05 & 0.04 & 0.05 & 0.02 & 0.96 \end{array}\right]$$

Scenario 5: Identify the class of targets within each unit with a type number M of 5. Three sensors are used to detect the targets. Add a sensor in addition to the one used in scenario 4. When the confusion matrix is set to a fixed value, the confusion matrix of the added sensor is as follows:

$$P^{7}=\left[\begin{array}{ccccc} 0.80 & 0.04 & 0.01 & 0.025 & 0.05\\ 0.05 & 0.84 & 0.01 & 0.025 & 0.05\\ 0.05 & 0.04 & 0.96 & 0.025 & 0.05\\ 0.05 & 0.04 & 0.01 & 0.900 & 0.05\\ 0.05 & 0.04 & 0.01 & 0.025 & 0.80 \end{array}\right]$$

The detection period of sensors in all scenarios is set to 1 s. Based on the above scenario information, analyze the sensor management efficiency for target recognition in different scenarios.

### 4.3. Simulation Results of the Proposed Algorithm

#### 4.3.1. The Process of Managing Single Sensors

Take the sensor recognition of two targets in scenario 1 as an example to illustrate the target recognition process after incorporating sensor management. To compare the proposed method with the methods based on information gain, discrimination, discrimination gain, and quadratic entropy under the same conditions, the true categories of targets 1 and 2 are fixed to 2 and 1, respectively. The initial recognition probability distribution of the fusion center for targets 1 and 2 is [0.5, 0.5]. The corresponding observations are generated in the simulation according to the true class of the target, rather than randomly based on the probability of occurrence of the measurement.

**Figure 4:**
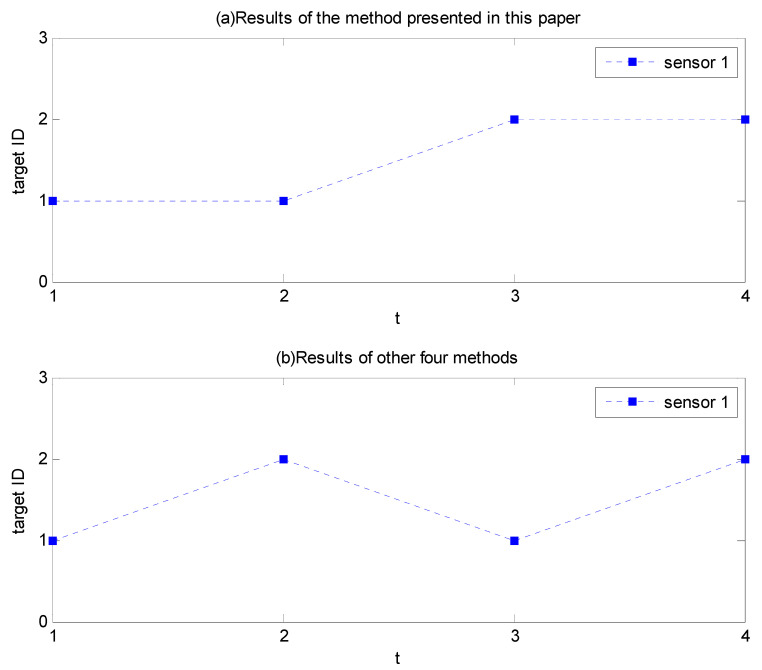
Processing of sensor recognition of 2 targets in scenario 1.

The specific processing process of the five methods is shown in Table 1, and the sensor assignment results are shown in Figure 4. Figure 4a shows the sensor assignment results of the methods in this paper, and Figure 4b shows the sensor assignment results based on the information gain, discrimination, discrimination gain, and quadratic entropy. It can be seen that after the sensor detects the target in the first second, the sensor assignment and identification results are the same for the five methods. However, in the second, since the information obtained by the detection of target 1 is smaller than that of target 2, the method based on information theory is chosen to detect target 2, while the proposed method continues to detect target 1. Thus, after two seconds, the proposed method makes target 1 confirmed recognition, while the other four methods cannot confirm the recognition of any target. Finally, all five methods can complete the recognition confirmation of two targets after four seconds, and the correct recognition rates are 100%, but the average recognition confirmation time of our method is 3 s, while the other methods are 3.5 s.

#### 4.3.2. The Process of Allocating Multiple Sensors

Assume that 10 targets are detected using three sensors in scenario 3. The proposed method is compared with the methods based on information gain, discrimination, discrimination gain, and quadratic entropy under the same conditions. The true categories of each of the 10 targets are fixed as follows: 3, 2, 2, 1, 3, 2, 2, 2, 2, and 1. The initial recognition probability distribution of the fusion center is the same for the 10 targets. The corresponding observations are generated in the simulation according to the true class of the targets, rather than randomly based on the probability of occurrence of the measurement.

$$\vec{\pi }\left(0\right)=\left[\begin{array}{c} \begin{array}{c} \begin{array}{ccc} 0.4197 & 0.1865 & 0.3938 \end{array}\\ \begin{array}{ccc} 0.3674 & 0.3998 & 0.2328\\ \;0.2637 & 0.7092 & 0.0271 \end{array}\\ \begin{array}{ccc} 0.3412 & 0.3520 & 0.3069\\ 0.1418 & 0.3763 & 0.4819\\ 0.4421 & 0.0888 & 0.4691 \end{array}\\ \begin{array}{ccc} 0.0851 & 0.5210 & 0.3939\\ 0.3249 & 0.2982 & 0.3769\\ 0.4631 & 0.1737 & 0.3632 \end{array} \end{array}\\ \begin{array}{ccc} 0.2034 & 0.0314 & 0.7652 \end{array} \end{array}\right]$$

**Figure 5:**
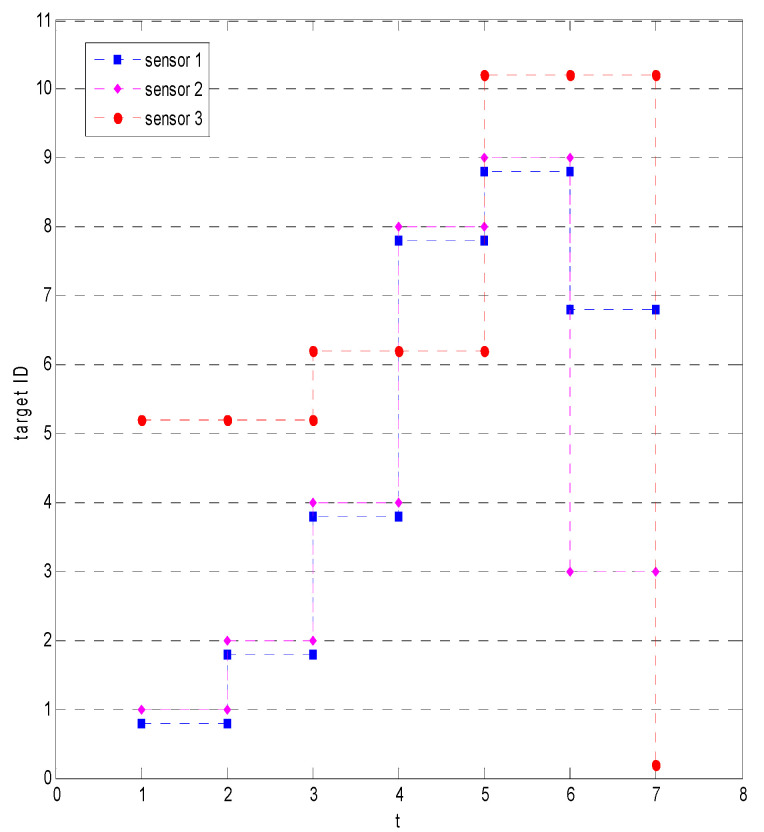
Sensor assignment results for the proposed method in scenario 3.

**Figure 6:**
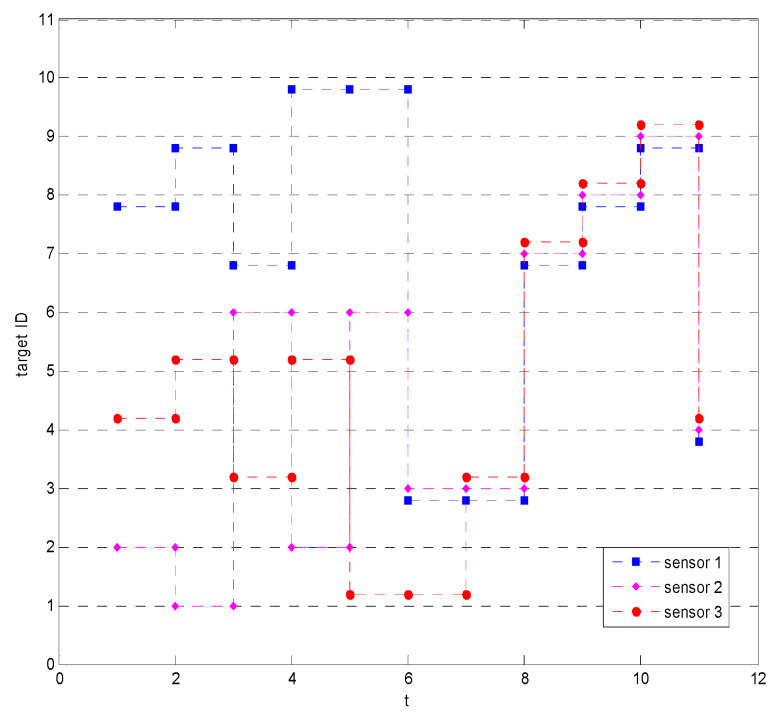
Results of sensor assignment based on information gain method in scenario 3.

The results of the proposed method and the results based on information gain are shown in Figure 5 and Figure 6, respectively. The horizontal axis in the figure is time, the vertical axis is the target number, and the three curves indicate the targets assigned to be detected by the three sensors at each moment, taking Figure 5 as an example, the target numbers detected by sensor 1 in seven cycles are 1, 2, 4, 8, 9, 7, and 7. It can be seen from Figure 5 and Figure 6 that the proposed method can continuously allocate two sensor resources to low uncertainty targets at multiple moments, so that the target can be quickly confirmed identified. Additionally, the proposed method does not allocate all three sensors to the same target. However, other methods allocate sensor resources to multiple targets evenly at the beginning, so as to maximize the total reduction in uncertainty for all targets but give up the concentration of resources to make the low uncertainty target reach the recognition degree as soon as possible, resulting in a long time to complete the identification of all targets. The comparison results show that the proposed method can allocate sensor resources appropriately and without waste. The performances of different algorithms are shown in Table 2. The correct recognition rate of the proposed method is 100%, which is better than or equivalent to other methods. The average recognition–confirmation time is 4.1 s, and the final target identification–confirmation time is 7 s, which are the shortest of all methods.

#### 4.3.3. The Performance Comparison of Algorithms in 5 Scenarios

After visualizing the processing of the five algorithms in the above two experiments, this experiment compares the performance of the algorithms under large batch random simulation. In each simulation experiment, the real category of the target is set randomly. In the simulation, the probability of the target belonging to each category is determined according to the real category of the target and the confusion matrix, and the observation value is generated randomly according to this. The confusion matrix of the sensor, the initial probability of target recognition by the sensor, and the initial probability of target recognition by the fusion center are three factors that have a great influence on the simulation results.

In this paper, there are three simulation methods for the sensor confusion matrix:

A1: Fixed values specified in Section 4.2;

A2: The confusion probabilities are all random values, and the probability of correct recognition of the target category is guaranteed to be no less than 0.7;

A3: The recognition confusion probability of a certain type of target by the sensor conforms to the binomial distribution B(n − 1, p), where n is the number of target types, and the parameter p of class n targets is selected out from the fixed disordered set [0.12, 0.91, 0.23, 0.7, 0.45].

There are three simulation methods for the initial probability of target recognition by the sensor, which are as follows:

B1 follows uniform distribution;

B2 follows random distribution;

B3 represents the prior knowledge with noise, that is, the probability of correct recognition is equiprobability plus a random value of 0~0.2, and the probability of other recognition confusion is equal.

There are two initial probabilities for the fusion center to recognize each target, which are:

C1 follows the random distribution. That is, the initial recognition probability of each target is a random value that does not exceed the recognition confirmation threshold.

C2 represents the prior knowledge with noise, that is, the probability of correct recognition is equiprobability plus a random value of 0~0.2, and the probability of other recognition confusion is equal.

Based on the simulation methods of the above three factors, this paper sets five simulation parameter conditions, as shown in Table 3.

Under the above five simulation parameter setting conditions, the five scenarios were simulated 1000 times.

**Figure 7:**
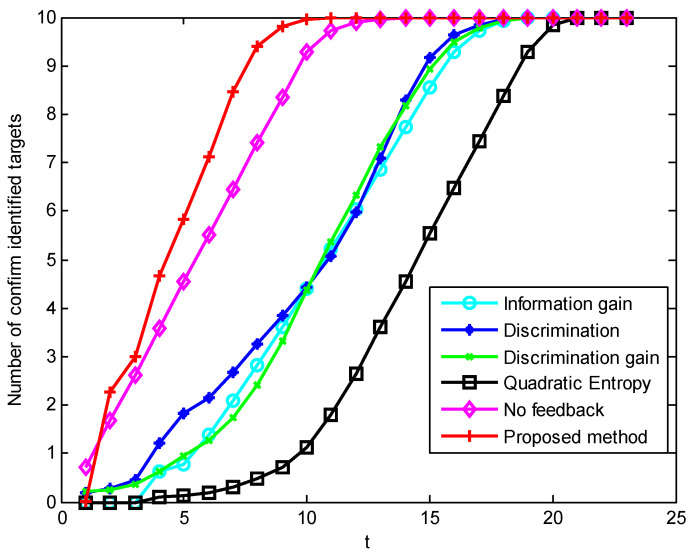
Sequence diagram of the number of confirmed targets in scenario 5 under condition 1.

**Figure 8:**
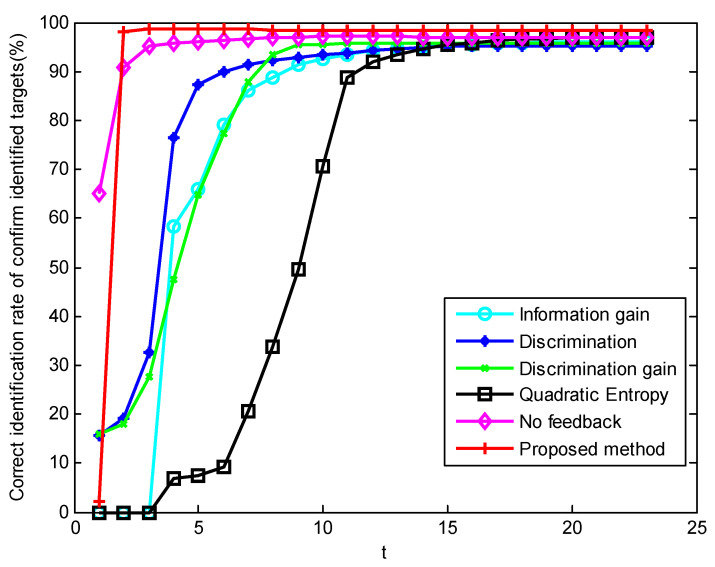
Sequence diagram of confirmed target recognition accuracy in scenario 5 under condition 1.

**Figure 9:**
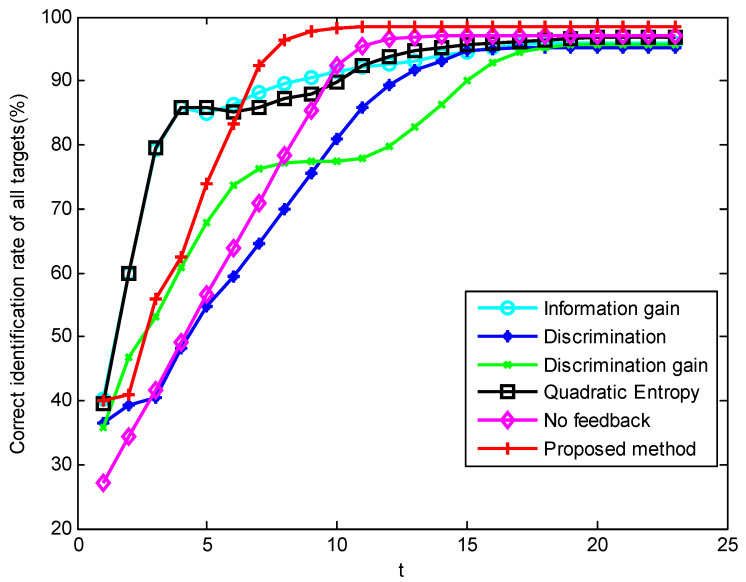
Sequence diagram of all target recognition accuracy rates in scenario 5 under condition 1.

**Figure 10:**
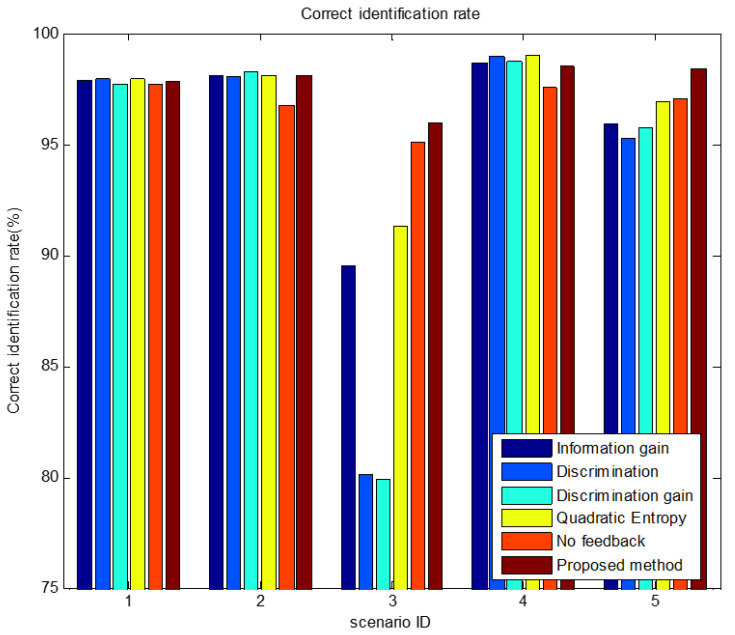
Comparison graph of correct recognition rate by various algorithms in scenario 5 under condition 1.

In scenario 5’s simulation of the first parameter setting, Figure 7, Figure 8 and Figure 9 show the performance changes of various algorithms with the advancement of simulation time. The horizontal axis of all three figures is time, measured in seconds. The vertical axis of Figure 7 is the number of targets that have completed recognition–confirmation at each time; the vertical axis of Figure 8 is the recognition accuracy rate of the identified targets at each time; and the vertical axis of Figure 9 is the recognition accuracy rate of all targets at each time, including the confirmed and unrecognized targets. It can be seen that in the early stage of the simulation, the method proposed in this paper has a large number of confirmed targets and a high correct recognition rate of identified targets, while the method based on information theory has a higher correct recognition rate of all targets. This shows that the method in this paper concentrates the sensor resources on the targets that can be identified, while the method based on information theory distributes the sensor resources equally to all targets. The correct recognition rate of different algorithms is shown in Figure 10. It can be seen that the correct recognition rate of the proposed method in this paper is comparable to or better than other methods.

**Figure 11:**
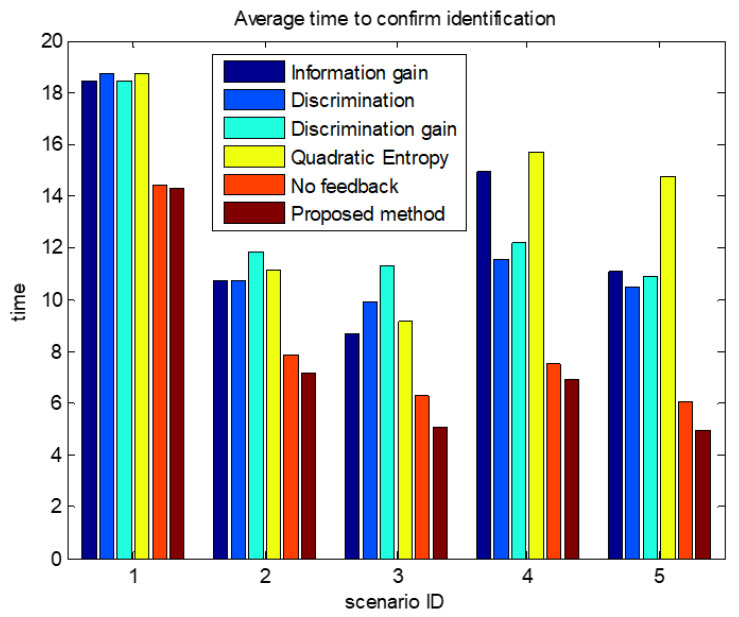
Comparison chart of average confirmation recognition time by various algorithms under condition 1.

**Figure 12:**
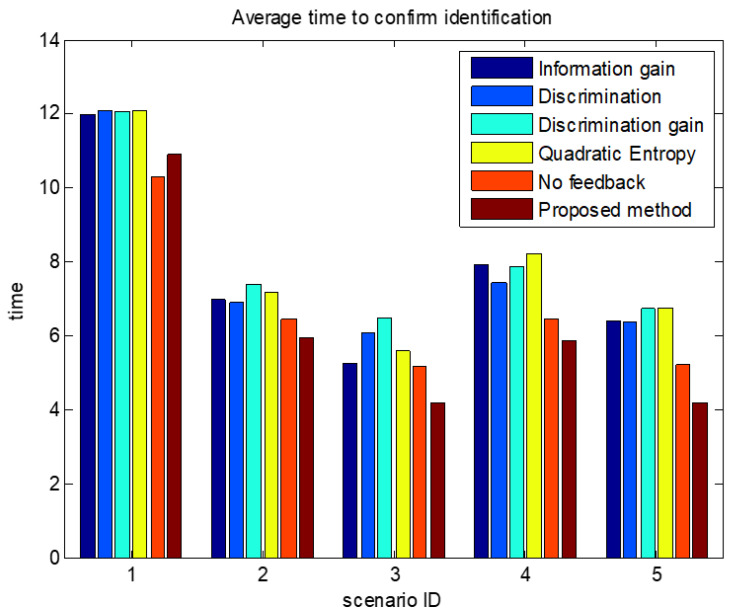
Comparison chart of average confirmation recognition time based on various algorithms under condition 2.

**Figure 13:**
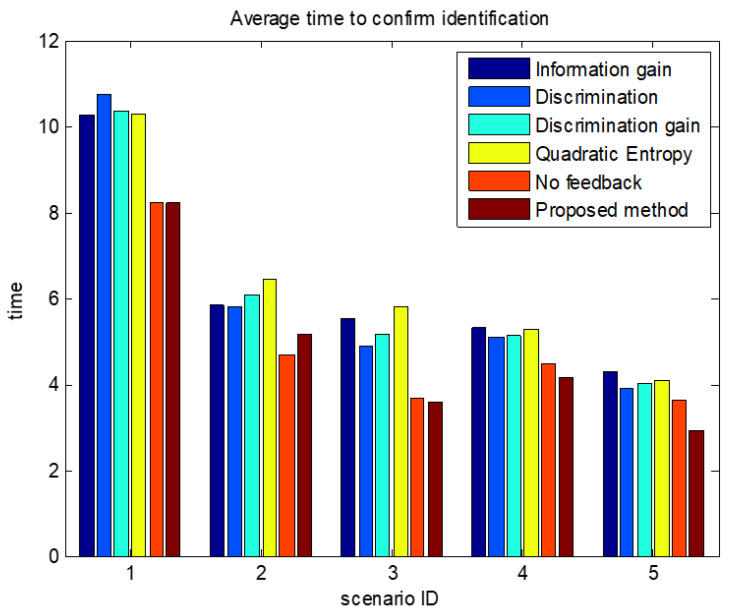
Comparison chart of average confirmation recognition time based on various algorithms under condition 3.

**Figure 14:**
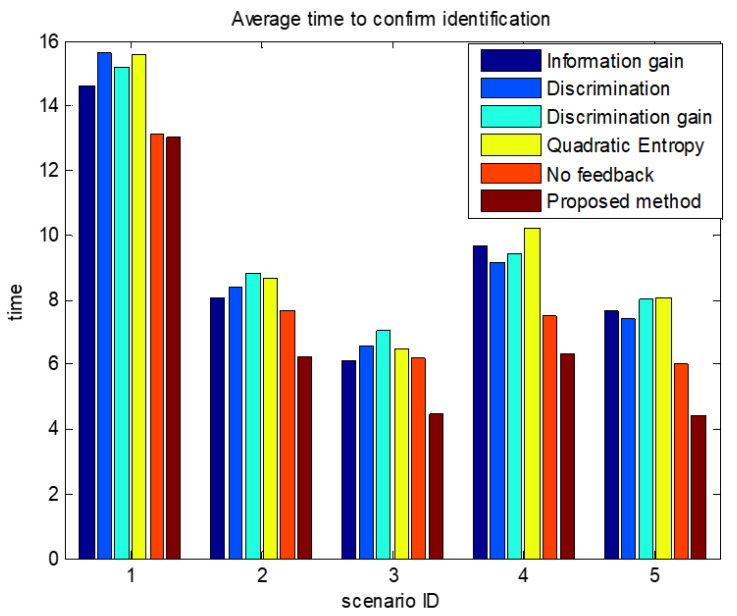
Comparison chart of average confirmation recognition time based on various algorithms under condition 4.

**Figure 15:**
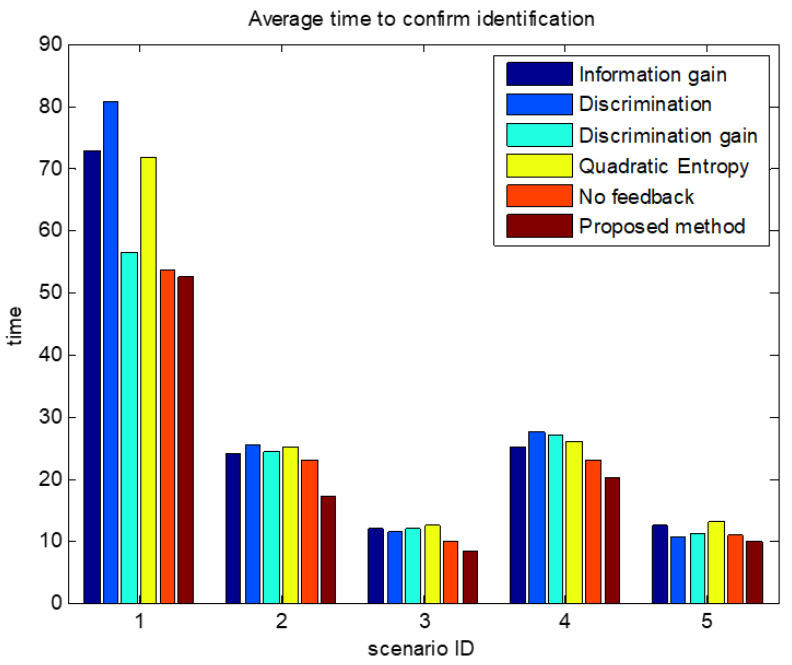
Comparison chart of average confirmation recognition time based on various algorithms under condition 5.

The comparisons of average confirmation recognition time based on various algorithms under the five simulation conditions are shown in Figure 11, Figure 12, Figure 13, Figure 14 and Figure 15. It can be seen that the average confirmation–recognition time of the proposed method is shorter than other methods in different scenarios, indicating that the proposed method achieves the purpose of shortening the target recognition–recognition time.

To verify the effectiveness of the proposed method that feedbacks the global identify results to local classifiers, we change the method of predicting the recognition result of basic sensors in step 3 of the target recognition process in Section 3.3 from Equation (14) to Equation (13), called the feedback-free confirmation recognition target-first sensor management method. It can be seen from Figure 11, Figure 12, Figure 13, Figure 14 and Figure 15 that the feedback-free confirmation recognition target-first sensor management method has inferior performance than the method in this paper, but it is better than the methods based on information gain, discrimination, discrimination gain, and quadratic entropy gain, which indicates that the effectiveness function based on information entropy and confirmed identification threshold is better, and the global fusion recognition result feedback local classifier target recognition prediction method can predict the recognition result more accurately, thus further shortening the recognition confirmation time.

## 5. Conclusions

A novel sensor management approach aimed at prioritizing the allocation of resources to identifiable targets is proposed in a hierarchical distributed target identification method. On the one hand, the method proposes to use the global recognition result feedback local classifier for the prediction of sensor recognition probability distribution in order to use the detection information of other sensors to improve the prediction accuracy of single-sensor recognition results and support a more reasonable allocation of sensors, which in turn reduces the recognition time. On the other hand, considering that information gain, discrimination, discrimination gain, and quadratic entropy gain are the measures of the amount of uncertainty change, while information entropy is a measure of recognition uncertainty that is directly related to the maximum probability in the predicted recognition result, it is more likely to improve the recognition speed under the target recognition with the maximum posterior criterion and recognition confirmation mechanism. The proposed method constructs a segmented effectiveness function based on the information entropy and target identification confirmation threshold, which can give more weight to targets meeting the identification confirmation threshold, thus facilitating the priority allocation of resources to confirmable identification targets. Compared with the methods based on information theory, the method proposed in this paper has a shorter average recognition confirmation time on the premise of ensuring the accuracy of target recognition and achieves the purpose of preferentially allocating resources to identifiable targets. At present, the method in this paper decides the sensor action in the next moment. Future work will determine how to extend this to the sensor management for a longer time.

## Figures and Tables

**Table 1:** Processing of sensor recognition of 2 targets in scenario 1.

		The Proposed Method	Information Gain	Discrimination	Discrimination Gain	Quadratic Entropy
Initial Status	Sensors	$\pi_{1}^{1}\left(0\right)=\left[0.5,0.5\right]$ $\pi_{2}^{1}\left(0\right)=\left[0.5,0.5\right]$	$\pi_{1}^{1}\left(0\right)=\left[0.5,0.5\right]$ $\pi_{2}^{1}\left(0\right)=\left[0.5,0.5\right]$	$\pi_{1}^{1}\left(0\right)=\left[0.5,0.5\right]$ $\pi_{2}^{1}\left(0\right)=\left[0.5,0.5\right]$	$\pi_{1}^{1}\left(0\right)=\left[0.5,0.5\right]$ $\pi_{2}^{1}\left(0\right)=\left[0.5,0.5\right]$	$\pi_{1}^{1}\left(0\right)=\left[0.5,0.5\right]$ $\pi_{2}^{1}\left(0\right)=\left[0.5,0.5\right]$
	Fusion Center	$\pi_{1}\left(0\right)=\left[0.5,0.5\right]$ $\pi_{2}\left(0\right)=\left[0.5,0.5\right]$	$\pi_{1}\left(0\right)=\left[0.5,0.5\right]$ $\pi_{2}\left(0\right)=\left[0.5,0.5\right]$	$\pi_{1}\left(0\right)=\left[0.5,0.5\right]$ $\pi_{2}\left(0\right)=\left[0.5,0.5\right]$	$\pi_{1}\left(0\right)=\left[0.5,0.5\right]$ $\pi_{2}\left(0\right)=\left[0.5,0.5\right]$	$\pi_{1}\left(0\right)=\left[0.5,0.5\right]$ $\pi_{2}\left(0\right)=\left[0.5,0.5\right]$
Second 1	Effectiveness Matrix q	$\left[\begin{array}{c} 0.2287\\ 0.2287 \end{array}\right]$	$\left[\begin{array}{c} 0.4426\\ 0.4426 \end{array}\right]$	$\left[\begin{array}{c} 0.4426\\ 0.4426 \end{array}\right]$	$\left[\begin{array}{c} 0.4426\\ 0.4426 \end{array}\right]$	$\left[\begin{array}{c} 0.6300\\ 0.6300 \end{array}\right]$
	Allocation results	Identify the target 1	Identify the target 1	Identify the target 1	Identify the target 1	Identify the target 1
	Sensor recognition results	$\left[\begin{array}{c} \begin{array}{cc} 0.13 & 0.87 \end{array}\\ \begin{array}{cc} 0.50 & 0.50 \end{array} \end{array}\right]$	$\left[\begin{array}{c} \begin{array}{cc} 0.13 & 0.87 \end{array}\\ \begin{array}{cc} 0.50 & 0.50 \end{array} \end{array}\right]$	$\left[\begin{array}{c} \begin{array}{cc} 0.13 & 0.87 \end{array}\\ \begin{array}{cc} 0.50 & 0.50 \end{array} \end{array}\right]$	$\left[\begin{array}{c} \begin{array}{cc} 0.13 & 0.87 \end{array}\\ \begin{array}{cc} 0.50 & 0.50 \end{array} \end{array}\right]$	$\left[\begin{array}{c} \begin{array}{cc} 0.13 & 0.87 \end{array}\\ \begin{array}{cc} 0.50 & 0.50 \end{array} \end{array}\right]$
	Fusion recognition results	$\left[\begin{array}{c} \begin{array}{cc} 0.13 & 0.87 \end{array}\\ \begin{array}{cc} 0.50 & 0.50 \end{array} \end{array}\right]$	$\left[\begin{array}{c} \begin{array}{cc} 0.13 & 0.87 \end{array}\\ \begin{array}{cc} 0.50 & 0.50 \end{array} \end{array}\right]$	$\left[\begin{array}{c} \begin{array}{cc} 0.13 & 0.87 \end{array}\\ \begin{array}{cc} 0.50 & 0.50 \end{array} \end{array}\right]$	$\left[\begin{array}{c} \begin{array}{cc} 0.13 & 0.87 \end{array}\\ \begin{array}{cc} 0.50 & 0.50 \end{array} \end{array}\right]$	$\left[\begin{array}{c} \begin{array}{cc} 0.13 & 0.87 \end{array}\\ \begin{array}{cc} 0.50 & 0.50 \end{array} \end{array}\right]$
Second 2	Effectiveness Matrix q	$\left[\begin{array}{c} 1.0000\\ 0.2287 \end{array}\right]$	$\left[\begin{array}{c} -0.0184\\ 0.4426 \end{array}\right]$	$\left[\begin{array}{c} 0.3407\\ 0.4426 \end{array}\right]$	$\left[\begin{array}{c} -0.1019\\ 0.4426 \end{array}\right]$	$\left[\begin{array}{c} -0.1615\\ 0.6300 \end{array}\right]$
	Allocation results	Identify the target 1	Identify the target 2	Identify the target 2	Identify the target 2	Identify the target 2
	Sensor recognition results	$\left[\begin{array}{c} \begin{array}{cc} 0.0218 & 0.9782 \end{array}\\ \begin{array}{cc} 0.5000 & 0.5000 \end{array} \end{array}\right]$	$\left[\begin{array}{c} \begin{array}{cc} 0.13 & 0.87 \end{array}\\ \begin{array}{cc} 0.87 & 0.13 \end{array} \end{array}\right]$	$\left[\begin{array}{c} \begin{array}{cc} 0.13 & 0.87 \end{array}\\ \begin{array}{cc} 0.87 & 0.13 \end{array} \end{array}\right]$	$\left[\begin{array}{c} \begin{array}{cc} 0.13 & 0.87 \end{array}\\ \begin{array}{cc} 0.87 & 0.13 \end{array} \end{array}\right]$	$\left[\begin{array}{c} \begin{array}{cc} 0.13 & 0.87 \end{array}\\ \begin{array}{cc} 0.87 & 0.13 \end{array} \end{array}\right]$
	Fusion recognition results	$\left[\begin{array}{c} \begin{array}{cc} 0.0218 & 0.9782 \end{array}\\ \begin{array}{cc} 0.5000 & 0.5000 \end{array} \end{array}\right]$	$\left[\begin{array}{c} \begin{array}{cc} 0.13 & 0.87 \end{array}\\ \begin{array}{cc} 0.87 & 0.13 \end{array} \end{array}\right]$	$\left[\begin{array}{c} \begin{array}{cc} 0.13 & 0.87 \end{array}\\ \begin{array}{cc} 0.87 & 0.13 \end{array} \end{array}\right]$	$\left[\begin{array}{c} \begin{array}{cc} 0.13 & 0.87 \end{array}\\ \begin{array}{cc} 0.87 & 0.13 \end{array} \end{array}\right]$	$\left[\begin{array}{c} \begin{array}{cc} 0.13 & 0.87 \end{array}\\ \begin{array}{cc} 0.87 & 0.13 \end{array} \end{array}\right]$
Second 3	Effectiveness Matrix q	[0.2287]	$\left[\begin{array}{c} -0.0184\\ -0.0184 \end{array}\right]$	$\left[\begin{array}{c} 0.3407\\ 0.3407 \end{array}\right]$	$\left[\begin{array}{c} -0.1019\\ -0.1019 \end{array}\right]$	$\left[\begin{array}{c} -0.1615\\ -0.1615 \end{array}\right]$
	Allocation results	Identify the target 2	Identify the target 1	Identify the target 1	Identify the target 1	Identify the target 1
	Sensor recognition results	$\left[\begin{array}{c} \begin{array}{cc} 0.0218 & 0.9782 \end{array}\\ \begin{array}{cc} 0.8700 & 0.1300 \end{array} \end{array}\right]$	$\left[\begin{array}{c} \begin{array}{cc} 0.0218 & 0.9782 \end{array}\\ \begin{array}{cc} 0.8700 & 0.1300 \end{array} \end{array}\right]$	$\left[\begin{array}{c} \begin{array}{cc} 0.0218 & 0.9782 \end{array}\\ \begin{array}{cc} 0.8700 & 0.1300 \end{array} \end{array}\right]$	$\left[\begin{array}{c} \begin{array}{cc} 0.0218 & 0.9782 \end{array}\\ \begin{array}{cc} 0.8700 & 0.1300 \end{array} \end{array}\right]$	$\left[\begin{array}{c} \begin{array}{cc} 0.0218 & 0.9782 \end{array}\\ \begin{array}{cc} 0.8700 & 0.1300 \end{array} \end{array}\right]$
	Fusion recognition results	$\left[\begin{array}{c} \begin{array}{cc} 0.0218 & 0.9782 \end{array}\\ \begin{array}{cc} 0.8700 & 0.1300 \end{array} \end{array}\right]$	$\left[\begin{array}{c} \begin{array}{cc} 0.0218 & 0.9782 \end{array}\\ \begin{array}{cc} 0.8700 & 0.1300 \end{array} \end{array}\right]$	$\left[\begin{array}{c} \begin{array}{cc} 0.0218 & 0.9782 \end{array}\\ \begin{array}{cc} 0.8700 & 0.1300 \end{array} \end{array}\right]$	$\left[\begin{array}{c} \begin{array}{cc} 0.0218 & 0.9782 \end{array}\\ \begin{array}{cc} 0.8700 & 0.1300 \end{array} \end{array}\right]$	$\left[\begin{array}{c} \begin{array}{cc} 0.0218 & 0.9782 \end{array}\\ \begin{array}{cc} 0.8700 & 0.1300 \end{array} \end{array}\right]$
Second 4	Effectiveness Matrix q	[1]	[−0.0184]	[0.3407]	[−0.1019]	[−0.1615]
	Distribution results	Identify the target 2	Identify the target 2	Identify the target 2	Identify the target 2	Identify the target 2
	Sensor recognition results	$\left[\begin{array}{c} \begin{array}{cc} 0.0218 & 0.9782 \end{array}\\ \begin{array}{cc} 0.9782 & 0.0218 \end{array} \end{array}\right]$	$\left[\begin{array}{c} \begin{array}{cc} 0.0218 & 0.9782 \end{array}\\ \begin{array}{cc} 0.9782 & 0.0218 \end{array} \end{array}\right]$	$\left[\begin{array}{c} \begin{array}{cc} 0.0218 & 0.9782 \end{array}\\ \begin{array}{cc} 0.9782 & 0.0218 \end{array} \end{array}\right]$	$\left[\begin{array}{c} \begin{array}{cc} 0.0218 & 0.9782 \end{array}\\ \begin{array}{cc} 0.9782 & 0.0218 \end{array} \end{array}\right]$	$\left[\begin{array}{c} \begin{array}{cc} 0.0218 & 0.9782 \end{array}\\ \begin{array}{cc} 0.9782 & 0.0218 \end{array} \end{array}\right]$
	Fusion recognition results	$\left[\begin{array}{c} \begin{array}{cc} 0.0218 & 0.9782 \end{array}\\ \begin{array}{cc} 0.9782 & 0.0218 \end{array} \end{array}\right]$	$\left[\begin{array}{c} \begin{array}{cc} 0.0218 & 0.9782 \end{array}\\ \begin{array}{cc} 0.9782 & 0.0218 \end{array} \end{array}\right]$	$\left[\begin{array}{c} \begin{array}{cc} 0.0218 & 0.9782 \end{array}\\ \begin{array}{cc} 0.9782 & 0.0218 \end{array} \end{array}\right]$	$\left[\begin{array}{c} \begin{array}{cc} 0.0218 & 0.9782 \end{array}\\ \begin{array}{cc} 0.9782 & 0.0218 \end{array} \end{array}\right]$	$\left[\begin{array}{c} \begin{array}{cc} 0.0218 & 0.9782 \end{array}\\ \begin{array}{cc} 0.9782 & 0.0218 \end{array} \end{array}\right]$
results	Target identification results	target 1: Identified correctly, Confirm recognition time: 2 s; target 2: Identified correctly, Confirm recognition time: 4 s	target 1: Identified correctly, Confirm recognition time: 3 s; target 2: Identified correctly, Confirm recognition time: 4 s	target 1: Identified correctly, Confirm recognition time: 3 s; target 2: Identified correctly, Confirm recognition time: 4 s	target 1: Identified correctly, Confirm recognition time: 3 s; target 2: Identified correctly, Confirm recognition time: 4 s	target 1: Identified correctly, Confirm recognition time: 3 s; target 2: Identified correctly, Confirm recognition time: 4 s
	Correct recognition rate	100%	100%	100%	100%	100%
	Average identification confirmation(s) time	3	3.5	3.5	3.5	3.5

**Table 2:** Performance comparison of sensor management algorithms.

Indicators	The Proposed Method	Based on Information Gain	Based on Discrimination	Based on Discrimination Gain	Based on Quadratic Entropy
Correct recognition rate (%)	100.0	100.0	80.0	90.0	100.0
Average confirmation recognition time (s)	4.1	6.9	11.6	12.6	7.6
Maximum confirmation recognition time (s)	7	11	19	18	12

**Table 3:** Simulation settings of parameters.

Condition	Confusion Matrix of Sensors	The Initial Probability of Target Recognition by the Sensor	The Initial Probability of Target Recognition by the Fusion Center
1	A1	B1	C1
2	A1	B2	C1
3	A1	B3	C2
4	A2	B1	C1
5	A3	B3	C2

## Data Availability

The confusion matrix of the sensor used to support the findings of this study are included within the article.

## Appendix A

For a certain target, *i*, to be identified, let q(x) is the probability density distribution of target state x before detection (prior distribution) and let p(x) be the estimated probability density distribution of state x after detection (posterior distribution). Here, the subscript *i* for distinguishing the target is omitted to simplify the description.

Information gain ΔI is defined as the prior entropy minus the posterior entropy, i.e.,

(A1) $$\Delta I\left(q\left(x\right);p\left(x\right)\right)=-\sum_{x=\omega_{1}}^{\omega_{M}}q\left(x\right)\log q\left(x\right)+\sum_{x=\omega_{1}}^{\omega_{M}}p\left(x\right)\log p\left(x\right)$$

If p(x)=q(x), then ΔI(q(x);p(x))=0, it means that this observation has not obtained any innovation. If p(x)≠q(x), then ΔI(q(x); p(x))≠0, it means that this observation has obtained innovation. In general, the posterior probability should not be less than the prior probability, so ΔI(q(x); p(x))≥0. In the target identification sensor management based on information gain, the entropy $H_{t}$ of the target state at time *t* and the mathematical expectation value $E\left[H_{t+1|t}\right]$ of entropy before the measurement at time *t* + 1 are calculated by a certain method first, and then the sensor resource allocation $\Delta I_{t}=H_{t}-E\left[H_{t+1|t}\right]$ is carried out according to the optimization principle of maximizing the sum $\Delta I_{t}$ of all targets [20].

The discrimination between p(x) and q(x) is defined as

(A2) $$D\left(p\left(x\right);q\left(x\right)\right)=\overset{\omega_{M}}{\underset{x=\omega_{1}}{\sum }}p\left(x\right)\log\left(p\left(x\right)/q\left(x\right)\right)$$

Discrimination is a measure of information. If p(x)=q(x), then D(p(x);q(x))=0, indicating that this observation did not provide any new information. if p(x)≠q(x), then if D(p(x);q(x))≠0, it shows that new information has been obtained from the observation. In general, the posterior probability should not be less than the prior probability, so D(p(x);q(x))≥0. After the observation at time *t*, let the posterior probability of target *i* be $\pi_{i}\left(t\right)$, and the posterior probability at time *t* + 1 is predicted to be $\pi_{i}\left(t+1|t\right)$, then the discrimination $D_{t+1|t}$ of $\pi_{i}\left(t+1|t\right)$ relative $\pi_{i}\left(t\right)$ can be calculated. The sensor management based on discrimination takes the maximum sum of all targets $D_{t+1|t}$ as the optimization principle for the sensor-target assignment [19].

The discrimination gain ΔD is defined as the difference between discrimination before and after observation.

(A3) $$\Delta D=E\left(D_{t+1|t}\right)-D_{t|t-1}$$

where $E\left(D_{t+1|t}\right)$ is the predicted expectation of the discrimination before observation at the time *t* + 1, $\mathrm{and}\;D_{t|t-1}$ is the discrimination after observation at time *t*. The sensor management method based on discrimination gain takes maximization the discrimination gain as the optimization principle to allocate sensor resources.

When parameter α is 2, the Rényi entropy is the quadratic entropy:

(A4) $$H_{R_{\alpha }}\left(X\right)=\frac{1}{1-\alpha }\log_{2}\underset{x\in \Psi }{\sum }p^{\alpha }\left(x\right)$$

The sensor management method based on quadratic entropy is based on the principle of maximizing the sum of expected information gain of all targets to allocate sensor resources. Where the expected information gain is the difference between the quadratic entropy $H_{R_{2}}^{t}$ at time *t* and the mathematical expected value $E\left[H_{R_{2}}^{t+1|t}\right]$ of the quadratic entropy before measurement at time *t* + 1:

(A5) $$\Delta I_{R_{2}}^{t}=H_{R_{2}}^{t}-E\left[H_{R_{2}}^{t+1|t}\right]$$
